# One quick and simple fixation method: posterior malleolus fractures in spiral tibial fractures

**DOI:** 10.1186/s12891-023-06319-8

**Published:** 2023-03-30

**Authors:** Hongfei Qi, Zhong Li, Teng Ma, Cheng Ren, Yibo Xu, Qiang Huang, Haoxuan Feng, Kun Zhang, Yao Lu, Ming Li

**Affiliations:** Department of Orthopaedics and Trauma, Hong Hui Hospital, Xi’an Jiaotong University College of Medicine, No. 555, East Youyi Road, Xi’an, 710000 Shaanxi People’s Republic of China

**Keywords:** PMF, Fixation, Screw, Retrospective study, Rehabilitations

## Abstract

**Objective:**

Spiral fracture of tibia combined with posterior malleolar fracture (PMF) is a special and regular injury. There is no uniform fixation method for PMF in this kind of injury. Intramedullary nail is the first choice for the treatment of tibial spiral fracture. We proposed a minimally invasive percutaneous screw combined with intramedullary nail technology to fix the PMF in the tibial spiral fracture. This study aims to explore the effectiveness and advantages of this technology.

**Materials and methods:**

From January 2017 to February 2020, 116 cases of spiral fracture of tibia combined with PMF who were operated in our hospital were divided into Fixation Group (FG) and No Fixation Group (NG) according to whether PMF was fixed. After minimally invasive percutaneous screw fixation of ankle fracture in FG patients, the tibial intramedullary nail was inserted to fix the fracture. Collected the operation and postoperative recovery of the two groups of patients, including the operation time, intraoperative blood loss, AOFAS score, VAS score and dorsiflexion restriction of ankle joint at the last follow-up, and compared whether there is any difference between the two groups of patients.

**Results:**

The fracture of both groups healed.2 patients in NG had secondary displacement of PMF during operation, and the fracture finally healed after fixation. There were statistical differences between the two groups in terms of operation time, AOFAS score and weight bearing time. The operation time of FG was 67.9±11.2 min, and that of NG was 60.8±9.4 min; The weight bearing time of FG was 57.35±34.72 days, and that of NG was 69.17±21.43 days; The AOFAS score of FG was 92.50±3.46, and that of NG was 91.00±4.16. There were no significant difference in blood loss, VAS and dorsiflexion restriction of ankle joint between the two groups. The blood loss of FG was 66.8±12.3 ml, the blood loss of NG was 65.6±11.7 ml, the VAS score of FG was 1.37±0.47, the VAS score of NG was 1.43±0.51, the dorsiflexion restriction of FG was 5.8±4.1; the NG was 6.1±5.7.

**Conclusion:**

For the injury of tibial spiral fracture combined with PMF, our fixation technology can achieve minimally invasive fixation of PMF with percutaneous screws on the basis of intramedullary nail fixation of tibial fracture, promoting early functional exercise of ankle joint and early weight bearing of patients. This fixation technology is also characterized by simple and fast operation.

**Supplementary Information:**

The online version contains supplementary material available at 10.1186/s12891-023-06319-8.

## Introduce

Tibial spiral fracture combined with posterior malleolar fracture is a regular combination, and posterior malleolar fracture(PMF)is usually hidden [1–3]. Bostman first reported that 0.6% of tibial shaft fractures were complicated by ankle fractures [4]; Van der Werken and Zeegers reported an incidence of 11.5% [5]. Hou et al. examined 288 patients with tibial spiral fractures and found that 16.7% had posterior malleolus fractures [2]. As mentioned above, as clinicians realize the strong correlation between tibial spiral fracture and posterior malleolus fracture and the popularization of CT and other examination technologies, more and more posterior malleolus fractures related to tibial spiral fracture are detected [6, 7], and at the same time, its treatment problems are gradually paid attention to.

Intramedullary nailing is the first choice for tibial fracture fixation [8, 9]. The patients have good tolerance, early bearing time, low reoperation rate and poor line of force [10]. However, posterior malleolus fractures are joint-site fractures, and single intramedullary nailing is not appropriate for fractures involving the periarticular and metaphyseal regions. In patients with posterior malleolus fractures, secondary displacement of the posterior malleolus fracture may occur during the insertion of intramedullary nailing [11–13]. There is also a risk of displacement of the posterior malleolus fracture mass during rehabilitation exercise and early weight bearing, which may require secondary surgery. A study by Harish Kempegowda et al. [11]. showed that PMF should be fixed before intramedullary nail is placed for the injury of tibia combined with posterior malleolus fracture. The fixation of posterior malleolar fracture includes open reduction plate and screw fixation or percutaneous screw fixation [14]. At present, there is no consensus on what kind of fixation should be used for posterior malleolar fracture in this kind of injury. Since most of the posterior malleolar fractures are not displaced [15, 16], percutaneous screw fixation may be a better choice. No matter which fixation method is selected, the internal fixation device for fixing the posterior malleolus should not interfere with the distal insertion of the intramedullary nail, and the trauma to the soft tissue should be minimized when cooperating with the treatment of tibial intramedullary nail.

In this study, we will introduce a technique for fixation of posterior malleolus fracture in tibial spiral fracture, which has the advantages of simple operation and small soft tissue damage, and the patients have obtained good clinical results.

## Patients and methods

### Inclusive criteria

1. 18 years < age < 70 years; 2.Diagnosed as fracture of the middle and lower 1/3 of the same tibia with PMF ; 3. No lower extremity vascular and neurological complications and hemiplegia.

### Exclusion criteria

(1) Bilateral lower limb fractures or ipsilateral lower limb fractures with other fractures; (2) Pilon fracture or internal malleolus fracture; (3) Open fracture or pathological fracture; (4) No impairment of ankle function and no serious ankle function disease before injury; (5) The time from injury to operation is more than 2 weeks.

This retrospective study was approved by the ethics committee of Xi’an Hong Hui Hospital, and the written informed consent of all participants was obtained. From January 2017 to February 2020, a total of 116 patients with middle-lower third of tibia fractures combined with PMF were admitted to Xi’an Hong Hui Hospital.

### Surgical techniques

#### No fixation Group

After the patient was satisfied with the anesthesia, used a tourniquet in the lower limbs. Usually, the infrapatellar approach was selected to place intramedullary nails to fix tibial fractures. The C-arm fluoroscopy machine determined the appropriate needle entry point. The ideal needle entry point was located at 9 mm outside the midpoint of the tibial platform in the anteroposterior perspective, and the lateral perspective was located at the front edge of the anterior joint slope. Then, drew and reduced the fracture, place the guide pin, and judged the fracture reduction and the position of the guide pin through fluoroscopy. The guide needle should be placed at the metaphysis of the distal tibia. After satisfactory fracture reduction, selected appropriate intramedullary nail for placement ; re fluoroscopy confirmed the reduction of the fracture. After confirmed that the fracture reduction was satisfactory, the distal and proximal screws were placed in turn. Closed the wound layer by layer after repeated flushing.

#### Fixation Group

**Fig. 1:**
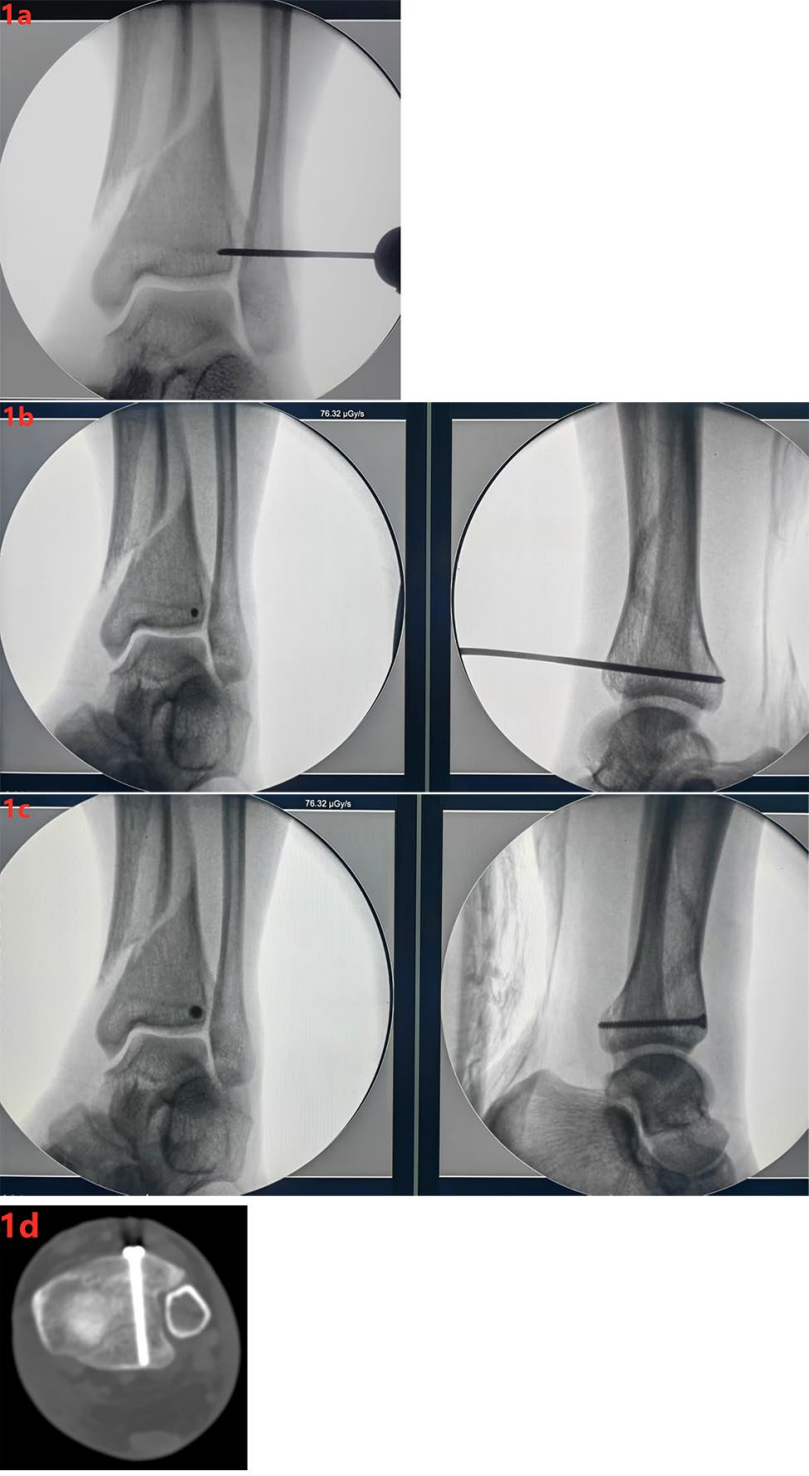
(**a**) X-ray films of standard ankle acupoints were projected by c-arm machine to locate the insertion point of screw channel; (**b**) Use a 2.5 mm Kirschner pin as the drill bit to drill the screw channel. After the position of the channel is confirmed by fluoroscopy, measure the length of the screw channel and tap; (**c**) Screw the screw into the channel for fixation (the channel direction is roughly parallel to the direction of the lower tibiofibular notch in the anteroposterior position, while in the lateral X-ray film, it should be consistent with the posterior inclination of the ankle joint); (**d**) Image of screw on CT cross section

The patient was in supine position and satisfied with general anesthesia or epidural anesthesia, used a tourniquet in the lower limbs. The C-arm fluoroscopy machine projected the X-ray film of the standard ankle acupoints, located the screw channel into the needle point, made a small skin incision, and stripped it to the bone surface of Chaput tubercle. The ideal channel opening should be located on the bone surface of the Chaput tubercle protrusion, about 5 mm below the epiphyseal line from the ankle joint surface and the lower tibiofibular notch. Used a 2.5 mm Kirschner wire to drill the screw channel (Fig. 1a). After the channel position was confirmed to be good by fluoroscopy, measured the length of the screw channel and tap. The direction of the channel drilling should be roughly parallel to the projection angle of the C-arm fluoroscopy machine, so that the direction of the channel was roughly parallel to the direction of the lower tibiofibular notch in the anterior posterior position, while in the lateral X-ray film, it should be consistent with the posterior inclination of the ankle joint (Fig. 1b). Finally, screws were inserted into the tunnel to fix the ankle fracture (Fig. 1c-d). The selection of screws can use cortical bone screws and hollow screws, which can be selected according to the specific situation: cortical bone screws, as position screws, are more applicable to the fracture of posterior malleolus crack without displacement, and their anatomical parts are maintained and fixed by position screws, which are usually directly screwed in after tapping. When the PMF fragments has a large separation and displacement, the hollow screw can be used as the lag screw for fixation. Before drilling the passage, it should be reset and temporarily fixed with a point clamp. After drilling the passage, inserted a guide pin and screwed the hollow screw along the guide pin. At this time, the hollow screw can be used to better achieve compression fixation between fracture ends. After fixation of PMF, tibial fracture was fixed by intramedullary nail, as in NG. Closed the wound layer by layer after repeated flushing.

### Postoperative treatment

No external fixation (plaster or brace, etc.) was performed in both groups. Antibiotics were given within 24 h after operation to prevent infection. Active and passive functional exercise of the ankle joint was started on the third day after operation. Non weight bearing was started on the fourth week after operation. Complete weight bearing was performed after imaging examination indicated that the fracture healed completely.

### Evaluation measures

The evaluation of the results of the study mainly included the operation and postoperative recovery. The patient’s medical records were obtained, mainly including the operation time and intraoperative blood loss. The postoperative recovery was evaluated by collecting the data of outpatient reexamination and telephone follow-up, mainly including the time of weight bearing, follow-up and the function of ankle joint at the last follow-up. The function of ankle joint is evaluated by AOFAS score, VAS score and ankle joint dorsiflexion restriction. The AOFAS questionnaire assessed pain (40 points), daily living function (28 points), range of motion (22 points) and ankle joint alignment (10 points) to reflect the function of the ankle joint. Higher score reflects better ankle function. The Visual Analog Scale (VAS) was used to quantify current pain (range 0: no pain to 100: intolerable pain). The restriction of ankle dorsiflexion is compared with the dorsiflexion of the healthy ankle, which can reflect the recovery of ankle motion function after surgery.

### Statistical analysis

Data analysis was performed using SPSS version 13.0. Continuous variables were reported as the mean±standard deviation. The continuous variables of two groups of patients were compared by two independent sample t-test or nonparametric test. Chi square test was used to compare the counting data. P < 0.05, the difference between the two groups was statistically significant.

## Results

The demographics data and radiological records were collected. There were 76 males and 40 females, ranging in age from 21 to 64 years, with an average age of 38.5 years. All patients completed X-ray and CT examinations before surgery, and were divided into fixation group (FG) and no fixation group (NG) according to whether the PMF was fixed or not. There were 60 cases in FG and 56 cases in NG. In terms of injury causes, 32 (51.6%) cases of FG were caused by traffic accidents, 18 (29.0%) cases were sprain, and 12 (19.4%) cases were caused by other causes; In NG, there were 28 (51.9%) traffic accident injuries, 16 (29.6%) sprains and 10 (18.5%) injuries caused by other reasons. The time from injury to operation was 4.71.3 days in FG and 4.31.1 days in NG. There were no statistically significant difference between the two groups in demographic data and preoperative general conditions (P < 0.05), as shown in Table 1.

**Table 1:** Comparison of demographic data and basic data between the two groups

Project	FG(n = 62)	NG(n = 54)	P
Age(years)	37.9±4.9	38.5±5.2	0.524
Sex			
Male (%)	40 (64.5)	36 (66.7)	0.808
Female (%)	22 (35.5)	18 (33.3)	
BMI	25.57±3.19	25.68±3.23	0.854
Cause of injury			
Traffic accident (%)	32 (51.6)	28 (51.9)	0.993
Sprain (%)	18 (29.0)	16 (29.6)	
Other (%)	12 (19.4)	10 (18.5)	
Percentage of articular surface involvement	35.9±12.7	35.4±10.7	0.821
Time from injury to operation(day)	4.7±1.3	4.3±1.1	0.079

All patients completed at least 12 months of follow-up, the follow-up time of FG was 14.3±2.6 months, and NG was 15.1±1.9 months. The fractures of both groups healed smoothly, and no patients reported serious complications such as internal fixation failure and revision surgery (Table 2). In NG, 2 (0.04%) patients had the displacement of posterior ankle fracture block during operation, and we fixed it in time. The operation time of FG was 67.9±11.2 min, and that of NG was 60.8±9.4 min. There was a statistical difference between the two groups (P < 0.05). The intraoperative blood loss of FG was 66.8±12.3ml, and that of NG was 65.6±11.7ml. There was no statistical difference between the two groups (P = 0.593).The weight bearing time of FG was 57.35±34.72 days, and that of NG was 69.17±21.43 days. There was a statistical difference between the two groups (P < 0.05).At the last follow-up, the AOFAS score of FG was 92.50±3.46 and that of NG was 91.00±4.16. There was a statistical difference between the two groups (P < 0.05), while the VAS of FG was 1.37±0.47 and that of NG was 1.43±0.51. There was no statistical difference between the two groups (P = 0.511).Although the two groups of patients had a certain degree of dorsiflexion restriction of ankle after surgery, the FG patients were 5.8±4.1, and the NG patients were 6.1±5.7, but there was no statistical difference between the two groups of patients (P = 0.743). Typical cases can be found in supplementary materials.

**Table 2:** Comparison of clinical results between FG and NG.

Project	FG(n = 62)	NG(n = 54)	P
Operation time(min)	67.9±11.2	60.89±9.4	< 0.001
Intraoperative blood loss(ml)	66.89±12.3	65.69±11.7	0.593
AOFAS scale	92.509±3.46	91.009±4.16	0.036
VAS	1.379±0.47	1.439±0.51	0.511
Weight bearing time(day)	57.359±34.72	69.179±21.43	0.032
Dorsiflexion restriction	5.89±4.1	6.19±5.7	0.743
Follow up time(month)	14.39±2.6	15.19±1.9	0.064

## Discussion

The middle and lower 1/3 spiral fracture of the tibia combined with ipsilateral PMF is a regular and special injury [17–20]. Because this type of PMF is different from simple PMF in etiology, injury mechanism, etc. [21, 22], its treatment method should also be different from ordinary PMF. At present, there is no standard treatment plan for this type of PMF. For the treatment of tibial shaft fracture, intramedullary nail has the advantages of good patient tolerance, early weight bearing time, low reoperation rate and low incidence of poor line of force [23]. Therefore, our research aims to solve how to achieve minimally invasive fixation of posterior ankle fracture on the basis of intramedullary nail treatment of tibial shaft fracture.

Simple posterior malleolus fracture is rare. Donken et al. [24]. showed that simple posterior malleolus fracture can be treated non operatively. Gardner et al. [25]. showed that surgical treatment of posterior ankle fracture can reduce the incidence of ankle joint complications. When the middle and lower 1/3 spiral fracture of the tibia is combined with posterior malleolus fracture, the posterior malleolus is mostly a hidden fracture, and the fracture line can’t be displayed under the ordinary X-ray, so it is easy to miss diagnosis [16]. Especially for the elderly patients with osteoporosis, minor trauma can cause fracture. Because of little violence, the fracture often does not shift [5]. In addition, osteoporosis leads to a decrease in bone density, and the fracture area is almost equal to the surrounding bone density. It is more difficult to identify the X-ray.

The indications for surgical treatment of PMF in tibial spiral fracture are controversial. The traditional view is that the size of PMF fragments is an important factor to determine whether surgical treatment, because the larger the size of PMF fragments, the worse the stability of ankle joint [26]. Many studies have shown that fractures with PMF greater than 25% of the tibial articular surface require surgical fixation [27]. Subsequent studies have shown that it may not be reliable to decide whether to perform surgical fixation only according to the size of the PMF fragments. The shape of the fracture, combined injury and injury mechanism also deserve attention for the selection of treatment plans [28]. Intramedullary nail is the preferred treatment for tibial shaft fracture [29]. For injuries associated with PMF, displacement of PMF fragments may occur during the insertion of intramedullary nail, which increases the incidence of ankle osteoarthritis. This risk also exists in the process of postoperative rehabilitation. At present, the main way to fix the PMF is plate or hollow screw fixation. The strength of plate fixation is greater than that of hollow screw fixation. Early ankle function exercise can be performed after surgery to reduce the incidence of fracture fragments displacement and restore ankle function at an early stage. Harish Kempegowda et al. [11]. suggested that PMF should be fixed before intramedullary nail is placed for the injury of tibial spiral fracture combined with PMF. The PMF pattern in spiral fractures of the tibia is simple. Most of them are triangular or shell shaped fragments in the posterolateral corner of the tibia, which do not extend to the medial malleolus [17, 30]. Most of the PMF in tibial spiral fractures are non displaced [15]. Although the plate can provide greater fixation strength, it may be excessive for such injuries. Compared with plate fixation, screw fixation has less damage to surrounding soft tissues and shorter operation time. For PMF without displacement or with small displacement in tibial spiral fractures, screw fixation may be more applicable. However, there is no unified view on the fixation of screws at that position and the fixation with several screws. The use of percutaneous screw fixation should avoid interfering with the distal placement of intramedullary nail, but there is no unified fixation method at present. Our center has summarized a set of methods of percutaneous screw fixation of posterior malleolar fractur PMF in tibial spiral fracture through long-term clinical practice and achieved good clinical results.

The results of this study show that better clinical results can be obtained by using our technology to fix the PMF, and the time of weight bearing is earlier. At the last follow-up, the AOFAS scale of FG was 92.509±3.46, and that of NG group was 91.009±4.16. The AOFAS scale of FG was superior to that of NG, with a statistically significant difference (P < 0.05). In FG, after percutaneous screw fixation of the PMF, the micro-movement of the PMF will be reduced during postoperative exercise and rehabilitation, which is beneficial to the patient’s better ankle joint function and the AOFAS score. The operation time of FG is longer than that of NG, which may be because FG needs an additional operation to fix the PMF. In our long-term clinical practice, we found that it doesn’t take much time to fix PMF after mastering this fixation technique. Since our PMF fixation is minimally invasive percutaneous fixation, there is no significant increase in intraoperative blood loss, which is consistent with our findings. The VAS of the two groups were at a low level, and there was no significant difference. It is well known that joint stiffness is the most common complication of joint fracture surgery, which may result in dorsiflexion restriction of the ankle joint. The occurrence of ankylosis may be related to the instability of ankle and the long-term postoperative immobilization. Patients with ankle fracture should do early functional exercises to avoid the occurrence of ankylosis. In our study, both groups had mild ankle dorsiflexion restriction. The weight-bearing time of FG (57.359±34.72 day) is earlier than that of NG (69.179±21.43 day), which may explain that after the fixation ofPMF, patients feel better about themselves, and clinicians are more confident to encourage patients to bear weight early.

The ideal screw channel should be from Chaput tubercle to Volkman tubercle, which is consistent with the angle of the notch of the lower tibiofibular. In the horizontal plane, the channel is close to the anterior and posterior and lateral bone cortex of the tibia, with good fixation and holding force. In addition, the PMF usually runs in the direction of anterior lateral oblique posterior medial, so the PMF fragments can be more fixed near the lower tibiofibular notch, so that the channel runs as long as possible in the PMF fragments, thus enhancing its fixation stability. The screw channel is designed to run through the anterior and posterior bone cortex of the distal tibia, and is close to the bone cortex of the lower tibiofibular notch and the subchondral bone of the ankle joint surface. It not only has excellent fixation and holding force, but also perfectly avoids the distal end of the intramedullary nail, and does not affect the placement of the intramedullary nail and the distal locking. In addition, the design makes the passage in the tibia as long as possible, and the shape based on the posterior malleolar fracture line can penetrate as many PMF fragments as possible, so as to increase the stability of fixation of PMF fragments. These characteristics make this technology also have advantages in elderly patients with osteoporosis fracture.

The main limitation of our study is that it is a single center retrospective study with a small sample size. In addition, the biomechanical study of this technique has not been carried out yet. In the follow-up study, we look forward to a multicenter, large sample randomized controlled study and biomechanical study. We believe that this technology is helpful for the fixation of PMF in tibial spiral fracture. It can achieve minimally invasive fixation of PMF on the basis of ensuring intramedullary nail fixation of tibial fracture.

## Conclusions

Spiral fracture of tibia combined with PMF is a regular injury. Our technology can achieve percutaneous minimally invasive screw fixation of PMF while intramedullary nail fixation of tibia fracture. The fixation effect is reliable, which can ensure early functional exercise of ankle joint after surgery and obtain better clinical results. At the same time, it is simple and fast to operate.

## Electronic supplementary material

Below is the link to the electronic supplementary material.

Supplementary Material 1

## Data Availability

The datasets used and/or analysed during the current study available from the corresponding author on reasonable request.
