# Supplementary material for: One quick and simple fixation method: posterior malleolus fractures in spiral tibial fractures
Source: BMC Musculoskelet Disord. 2023 Mar 30;24:244. doi: 10.1186/s12891-023-06319-8 (PMC10061987; doi:10.1186/s12891-023-06319-8)

**The following is our supplementary introduction to this study:**

In clinical practice, for the injury of tibial spiral fracture combined with posterior malleolar fracture, the posterior malleolar fracture was not fixed before the intramedullary nail was placed, which may lead to the secondary displacement of posterior malleolar fracture, which may increase the risk of ankle osteoarthritis.

The following is a case of secondary displacement of posterior ankle fracture during operation (Fig.1a-c). First of all, we can see that there is no obvious displacement of posterior malleolus fracture during the insertion of intramedullary nail guide pin; after reaming, the posterior malleolus fracture was displaced during the placement of the main nail; Finally, we fixed the posterior malleolus fracture with screws after reduction.


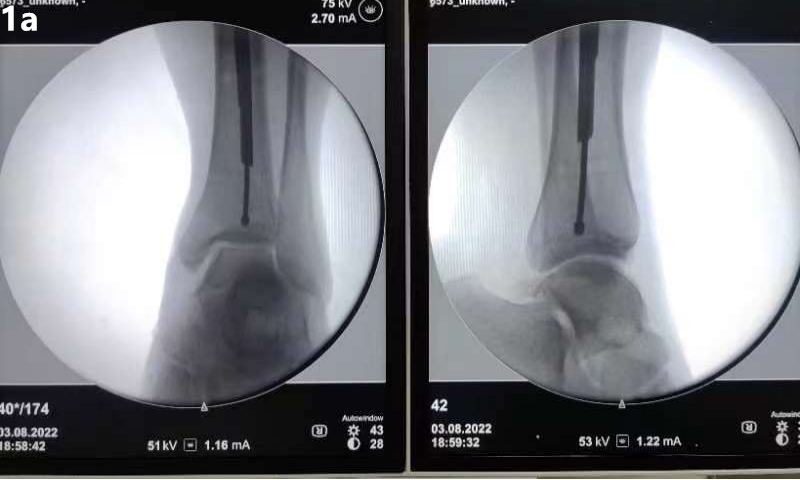


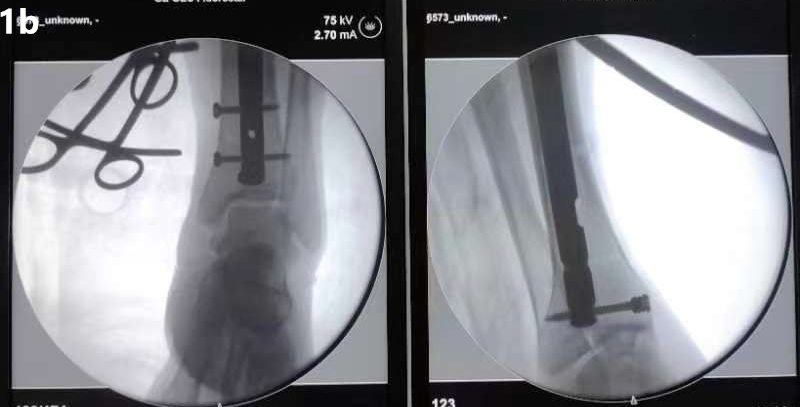


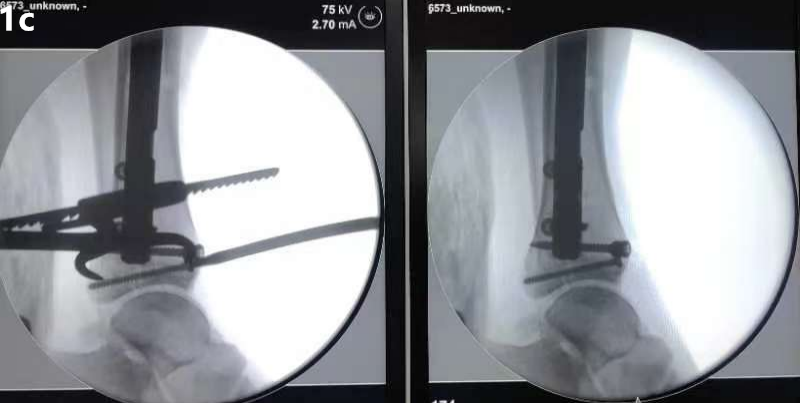


Fig. 1. **a:** There is no obvious displacement of posterior malleolus fracture during the insertion of intramedullary nail guide pin; **b:**The posterior malleolus fracture was displaced during the placement of the main nail; **c:**We fixed the posterior malleolus fracture with screws after reduction.

Our fixation technology can achieve minimally invasive fixation of posterior malleolus fracture, which depends on the characteristics of such posterior malleolus fracture injuries. They are usually non displaced, which can also explain why it is easy to miss the diagnosis of posterior malleolus fracture associated with tibial spiral fracture only depending on the suture.The ideal screw passage should be from Chaput tubercle to Volkman tubercle, which is consistent with the angle of the notch of the lower tibiofibular.In the horizontal section, the channel is close to the anterior and posterior and lateral bone cortex of the tibia, with good fixation and holding force. In addition, the posterior malleolus fracture line usually runs in the direction of anterior lateral oblique posterior medial, so the posterior malleolus bone block can be more fixed near the lower tibiofibular notch, so that the channel runs as long as possible in the posterior malleolus fracture block, thus enhancing its fixation stability.In the sagittal section, the ideal channel should be located below the epiphyseal line of the tibia, consistent with the retroversion angle of the ankle joint, and close to the area of the subchondral bone of the ankle joint to obtain better screw holding and fixation force.The screw channel is designed to run through the anterior and posterior bone cortex of the distal tibia, and is close to the bone cortex of the lower tibiofibular notch and the subchondral bone of the ankle joint surface. It not only has excellent fixation and holding force, but also perfectly avoids the distal end of the intramedullary nail, and does not affect the placement of the intramedullary nail and the distal locking. In addition, the design allows the passage to travel as long as possible in the tibia, and the shape based on the posterior malleolar fracture line can penetrate as many posterior malleolar fracture blocks as possible to increase the stability of fixation of posterior malleolar fracture blocks.

Typical case: A 41 year old female patient with left tibia fracture and posterior malleolus fracture due to falls underwent surgical treatment. During the operation, minimally invasive screws were used for percutaneous fixation of posterior malleolus fracture and intramedullary nails were used for fixation of tibia fracture (Fig.2a-f). The surgical process was smooth and the patient had a good prognosis.


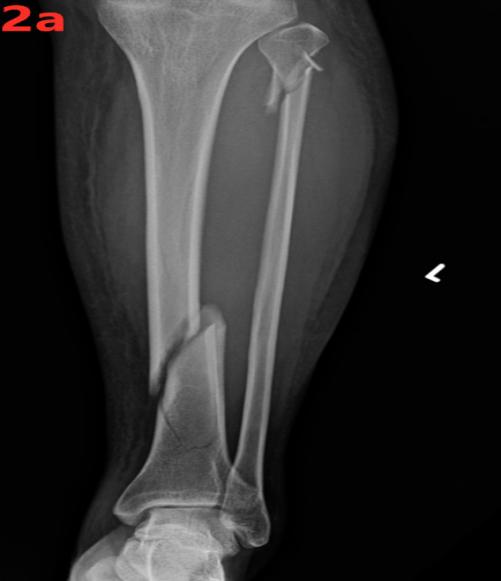

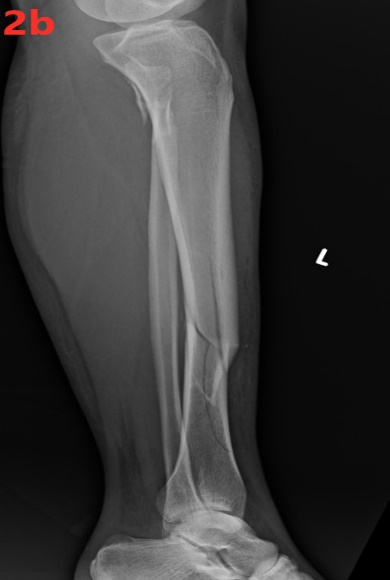

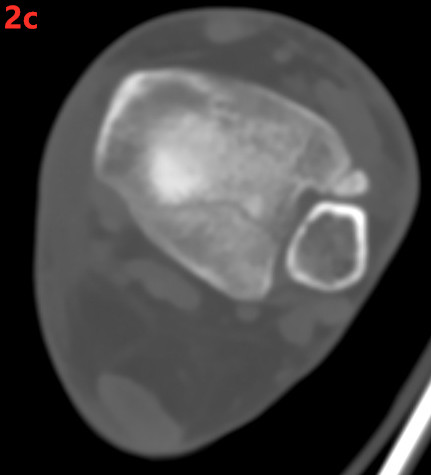


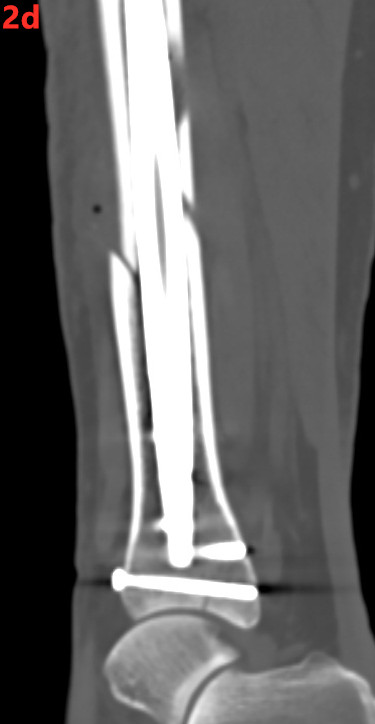

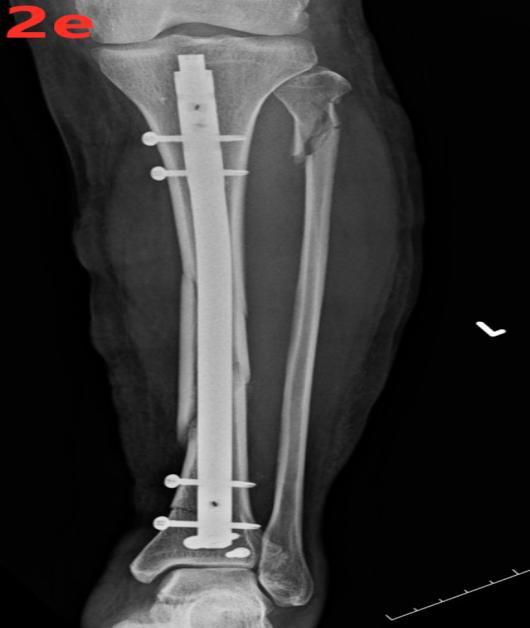

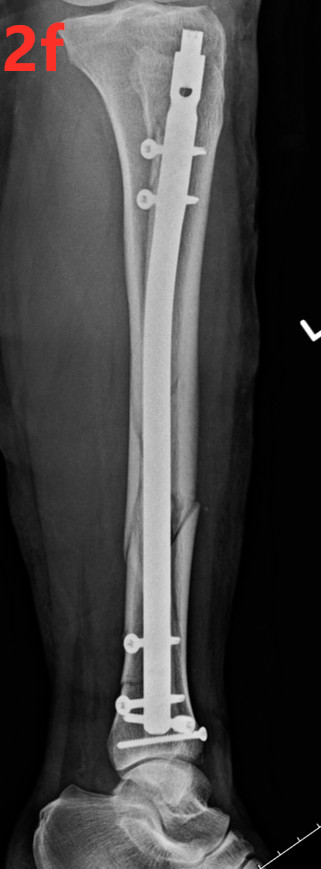


**Figure 2**. a-b. anterior and lateral X-ray film before operation; c. preoperative CT showed posterior malleolus fracture; d. postoperative three-dimensional CT showed good fixation of posterior malleolus fracture; e-f. postoperative anteroposterior and lateral X-ray films.

Previous fracture morphology studies have shown that,most of them are triangular or shell shaped fragments in the posterolateral corner of the tibia, which do not extend to the medial malleolus (Fig. 3).


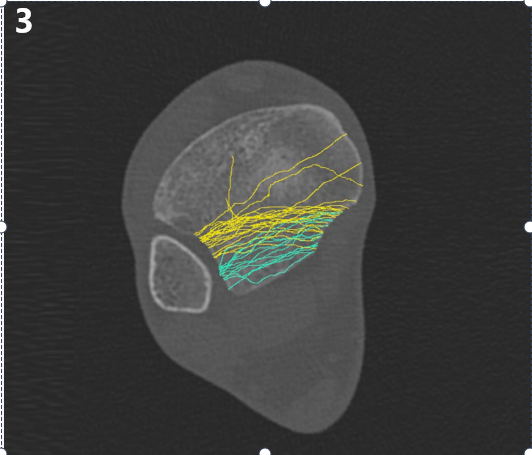

Supplement: Supplementary file 1 — Supplementary Material 1 [file 12891_2023_6319_MOESM1_ESM.docx]
